# Study on the effect of 40 Hz non-invasive light therapy system. A protocol for a randomized, double-blinded, placebo-controlled clinical trial

**DOI:** 10.3389/fnagi.2023.1250626

**Published:** 2023-10-12

**Authors:** Mikkel Pejstrup Agger, Maibritt Horning, Marcus Schultz Carstensen, Else Rubæk Danielsen, Anders Olhues Baandrup, Mai Nguyen, Peter Høgh, Kamilla Miskowiak, Paul Michael Petersen, Kristoffer Hougaard Madsen, Troels Wesenberg Kjær

**Affiliations:** ^1^Department of Neurology, Zealand University Hospital, Roskilde, Denmark; ^2^Department of Clinical Medicine, Faculty of Health and Medical Sciences, University of Copenhagen, Copenhagen, Denmark; ^3^Department of Electrical and Photonics Engineering, Technical University of Denmark, Kongens Lyngby, Denmark; ^4^Department of Radiology, Zealand University Hospital, Roskilde, Denmark; ^5^OptoCeutics ApS, Copenhagen, Denmark; ^6^Neurocognition and Emotion in Affective Disorders (NEAD) Centre, Psychiatric Centre Copenhagen, Copenhagen University Hospital, Rigshospitalet, Copenhagen, Denmark; ^7^Department of Psychology, Faculty of Social Sciences, University of Copenhagen, Copenhagen, Denmark; ^8^Department of Applied Mathematics and Computer Science, Technical University of Denmark, Kongens Lyngby, Denmark

**Keywords:** Alzheimer’s disease, gamma entrainment, 40 Hz, light-based neurostimulation, Invisible Spectral Flicker

## Abstract

**Introduction:**

With no cure or effective treatment, the prevalence of patients with Alzheimer’s disease (AD) is expected to intensify, thereby increasing the social and financial burden on society. Light-based 40 Hz brain stimulation is considered a novel treatment strategy for patients with AD that may alleviate some of this burden. The clinical trial ALZLIGHT will utilize a novel Light Therapy System (LTS). The LTS uses Invisible Spectral Flicker for non-invasive induction of 40 Hz neural activity. This protocol describes a trial evaluating the efficacy and safety of a light-based 40 Hz brain stimulation in patients with mild-to-moderate AD.

**Methods:**

62 patients with mild-to-moderate AD will participate in a randomized, double-blinded, placebo-controlled, parallel-group, and single-center trial. The participants will partake in an enrollment period of 1 month, an intervention period of 6 months, and a 1.5-month post-interventional follow-up period. Prior to the baseline measurement (week 0), the patients will be randomized to either active or placebo intervention from baseline (week 0) to post-intervention follow-up (week 26).

**Discussion:**

This protocol describes a randomized, double-blinded, placebo-controlled clinical trial that may increase the understanding of the effect of gamma oscillations in the human brain and how it could be utilized as a novel and important tool for the treatment of AD. The effect is measured through a large, multidisciplinary assessment battery.

**Clinical trial registration:**www.ClinicalTrials.gov, (NCT05260177). Registered on March 2, 2022.

## 1. Introduction

Alzheimer’s disease is the most common type of dementia with an enormous disease burden that is expected to impact over 40 million patients globally (Nichols et al., 2019). Early this year, the FDA granted accelerated approval for Lecanemab (FDA U.S. Food and Drug Administration, 2023), an amyloid-modifying therapeutic agent that was found to reduce cognitive decline in early AD patients by 27% over the 18 months of treatment (Reardon, 2023; van Dyck et al., 2023). Even though this is an important progression in finding treatment options, the approval has not been without controversy, and the safety, costs, and clinical relevance to patients and caregivers are discussed (The Lancet, 2022; Reardon, 2023). Amyloid-modifying therapeutic agents were found to carry the risk of causing Amyloid-related imaging abnormalities (ARIA) with edema or effusions (Sperling et al., 2011; Cogswell et al., 2022), which are reported with Lecanemab (Reardon, 2023; van Dyck et al., 2023). Consequently, new and safe treatment options are still needed.

Alternatively, the induction of gamma activity has been proposed as a possible novel approach to treating AD by exposure to flickering lights (McDermott et al., 2018; Adaikkan and Tsai, 2020). This idea leans on mice studies indicating that exposure to 1 h daily 40 Hz stroboscopic light leads to a reduction of Aβ, tau protein (Iaccarino et al., 2016; Adaikkan et al., 2019), and even improved visuospatial memory when combined with 40 Hz auditory stimulation (Martorell et al., 2019). The proposed mechanism is not perfectly understood, but it indicates that gamma entrainment directly affects GABAergic interneurons and leads to activation and/or recruitment of microglia, resulting in the reduction of Aβ and tau protein (McDermott et al., 2018; Singer et al., 2018; Adaikkan and Tsai, 2020). Similar mechanisms are observed in studies using Transcranial Magnetic Stimulation (TMS) which also causes increased gamma activation (Barr et al., 2009). Thus, the induction of gamma activity is a promising new approach to the treatment of AD.

Entrainment of a specific frequency of neural oscillations by exposure to stroboscopic light at the same frequency is well established (Herrmann, 2001). It is even used in routine electroencephalography (EEG) with stable reactions with increasing age (Kasteleijn-Nolst Trenité et al., 2012; Zibrandtsen et al., 2020).

Studies in patients with AD using non-invasive light for 40 Hz entrainment remain limited. Previous light-based studies used stroboscopic light for entrainment and either no sham or a sham of non-flickering white light with no blinding of the study participants for obvious reasons (Ismail et al., 2018; Hajos et al., 2020; Chan et al., 2021; Cimenser et al., 2021). The importance of blinding procedures is well established. It is essential when subjective measures are used, such as in scoring symptoms by either the patient, the caregivers, or study personnel (Day, 2000).

This study will use a novel form of light called Invisible Spectral Flicker (ISF) (Carstensen et al., 2020). ISF alternates between two color profiles to achieve a 40 Hz flicker which is perceived as uniformly non-flickering by the observer, as ongoing studies in our group investigate. Therefore, the participants, the caregivers, the study personnel, and the data analysts can all be blinded throughout the study.

In conclusion, the objective of the trial is to investigate the effect of 40 Hz non-invasive ISF stimulation and assess the possibility of ISF as a novel treatment option for AD.

## 2. Methods

### 2.1. Aim, study design, and setting

**Figure 1:**
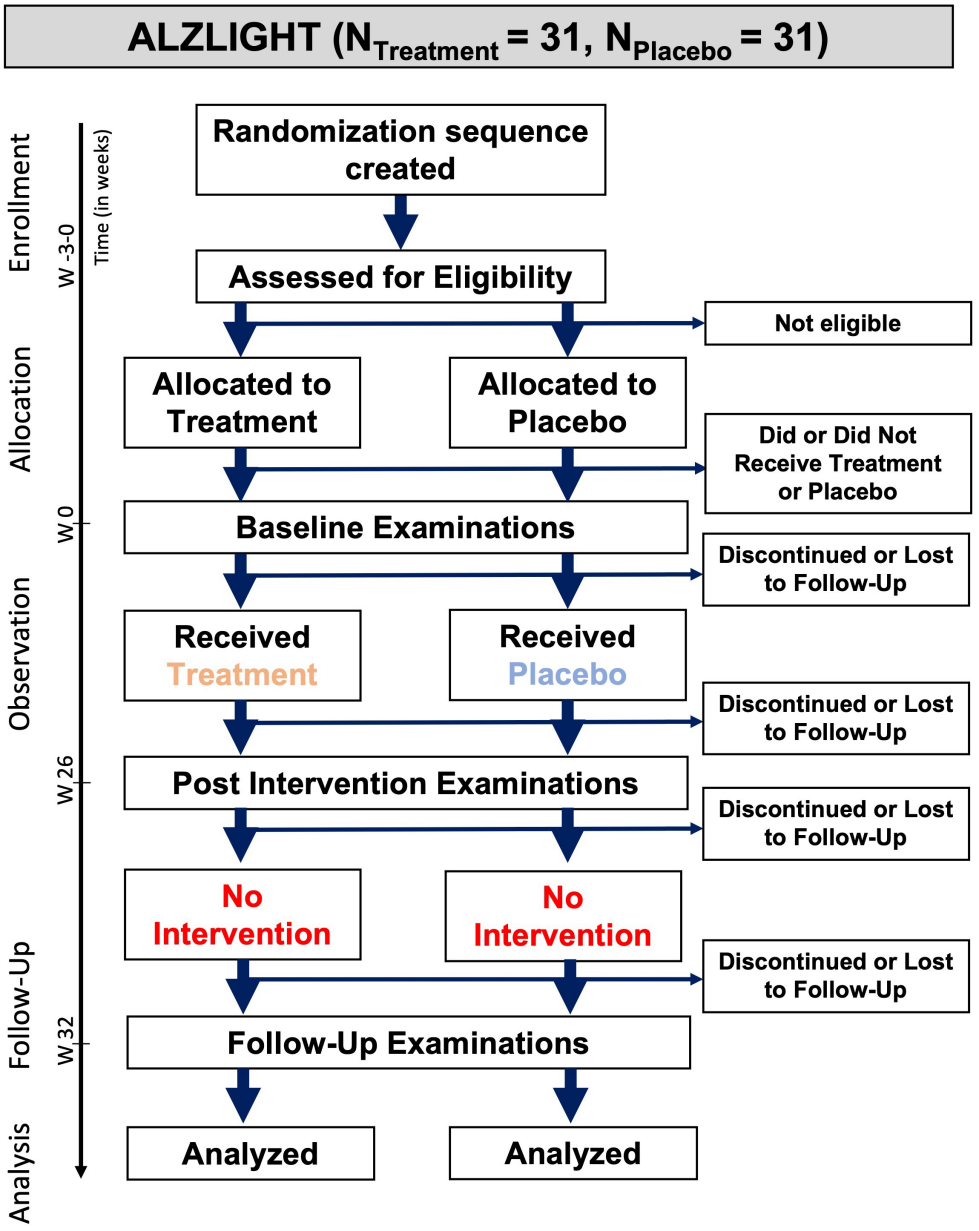
Flowchart of the study design.

This study will examine whether the entrainment of 40 Hz neural oscillation by novel 40 Hz Invisible Spectral Flicker is a potential therapy for AD. The study design will be a randomized, double-blinded (investigator, participant), placebo-controlled clinical trial. One arm of the study will receive active treatment with 40 Hz ISF, while the other arm will receive a placebo consisting of an identical sham device with non-flickering white light. The study will be conducted at the Zealand University Hospital, which is host to the regional memory clinic and the main diagnostic institution for neurological disease in the region of Zealand with an approximate population of 842.000 people (Danmarks Statistik, 2021). The intervention will last for 6 months (26 weeks) followed by 6 weeks of no intervention, see Figure 1. Those 6 weeks are designed to assess whether any potential effects are persisting after acute treatment.

### 2.2. Participants

This study will recruit a minimum of 62 patients with mild to moderate AD. Recruitment will continue until each arm reaches the target of 31 included participants. The participants must be above the age of 55, provide informed consent and match the following in- and exclusion criteria.

Inclusion criteria:

– Diagnosed with probable mild to moderate AD based on the National Institute on Aging and Alzheimer’s Association (NIA-AA) criteria (Jack et al., 2018)
– Speak Danish fluently
– >8 years of education
– Pass a color-blindness test (Ishihara color test)
– Have visual and auditory capabilities, and language skills necessary for neuropsychological testing
– Participants must have a designated caregiver, who is available to support the participant in complying

Exclusion criteria:

– Visual acuity >0.5
– Significant abnormalities related to relevant parts of the brain, e.g., the visual system, prefrontal cortex, or hippocampus, or relevant lesions detected by pre-trial imaging
– Prior history of diseases related to the brain or the visual system (excluding AD)
– Use of antiepileptic medication, neuromodulatory medication or high doses of sedatives
– History of substance abuse within the last 2 years
– Any significant systemic illness or unstable medical condition, which could lead to difficulty complying with the protocol (at the discretion of the PI)

### 2.3. Intervention

**Figure 2:**
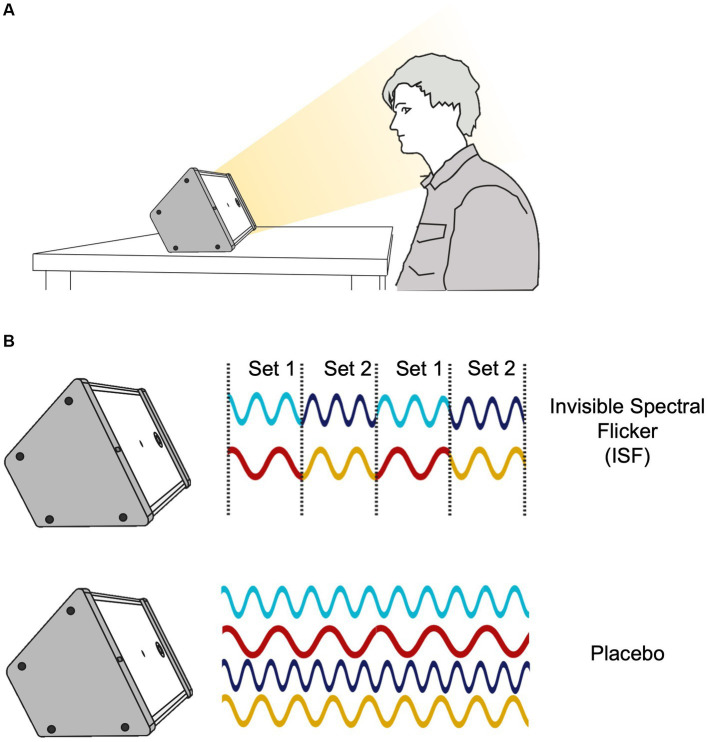
Study Intervention. **(A)** Intervention setup during daily stimulation. The participants are instructed to sit in front of the device at an arm’s length distance (50-100 cm distance). **(B)** Mechanism of the active intervention using Invisible Spectral Flicker (ISF) alternating between Set 1 and Set 2 and of the placebo intervention.

In both arms of this trial, participants will be instructed to sit in front of a specialized light source (henceforth “the device”) for 1 h daily during the intervention period of 6 months. The device must be within arm’s reach (at 50–100 cm distance), and the participants are instructed to face the device, see Figure 2A. The treatment group will have the device programmed to deliver 40 Hz ISF (Figure 2B), whilst the placebo group will have a sham device programmed to deliver non-flickering white light (Figure 2B) at the same intensity (~150 lux at the level of the eye) and color temperature (3,191 K, chromaticity coordinates of (x,y) = (0.410, 0.376)) as the treatment arm, equal to ISF and CONT in Agger et al. (2022). The devices used in this study will be the LTS 1.1 (Optoceutics ApS), the active setting in the device is equal to what is used in EVY LIGHT^®^ and NSS^®^ (Optoceutics ApS).

### 2.4. Blinding and randomization

The light device can be set to either ISF or sham, meaning that there is no visual difference between the devices used for active treatment or the sham devices used for placebo. Thus, neither the participant, the caregivers nor the investigators will be able to differentiate the devices and can, therefore, remain blinded throughout the study.

Allocation of study participants will be done by a prefabricated random allocation list, created by a biostatistician which allocates consecutively enrolled participants to group A or group B. A or B corresponds to either treatment or sham, determined by the biostatistician through a flip of a coin. This assignment will be kept separate from the allocation list. When all data are recorded, the investigator will be informed of the allocation A or B, but not of which are sham or active treatment, which will only be revealed upon completion of group-level analysis. The principal investigator will have an allocation list available to be used in case of serious adverse events (SAE’s) to assess whether the SAE has consequences for the continuation of the study.

### 2.5. Measurements

At the inclusion visit (week −3 to 0), after giving informed consent, the following baseline characteristics will be recorded during a pre-screening: Age (years), gender (male/female), ethnicity (White, black, or others), height (cm), weight (kg), alcohol use in units (one unit defined as 12 g pure alcohol), tobacco use (package years), time since diagnosis of AD (months) and education (years). The participants will further have their medical history reviewed including current medication, physical examination, and blood samples (plasma-Alat, plasma-HbA1C, plasma-Sodium, plasma-Potassium, and plasma-Creatinine), this is done to rule out any undiagnosed diseases and establish baseline values in case of adverse events. To assess the effects of the treatment, the following examination battery will be administered at baseline (week 0), post-intervention follow-up (week 26), and no-intervention follow-up (week 32): EEG steady state visual evoked potential (SSVEP) during exposure to the allocated device (not at week 32), resting-state electroencephalography (rs-EEG) (Cassani et al., 2018), Alzheimer’s Disease Assessment Scale-Cognitive Subscale Plus Executive Function & Functional Ability (ADAS-Cog Plus EF&FA) (Skinner et al., 2012), Alzheimer’s Disease Cooperative Study – Activities of Daily Living (ADCS-ADL) (Galasko et al., 1997), Montreal Cognitive Assessment (MoCA) (Nasreddine et al., 2005), Magnetic resonance (MR) imaging (high-resolution T1-weighted imaging, functional Blood Oxygen Level Dependent imaging (BOLD-fMRI), perfusion-weighted MR imaging and MR spectroscopy) and blood-based biomarkers (plasma Aβ42/40-ratio, plasma tau181, plasma tau231, plasma neurofilament light chain, and plasma glial fibrillary acidic protein). In addition to these time-specific measurements, continuous actigraphy will be collected by a wrist-worn actigraph to assess activity and changes in the circadian rhythm. All data will be recorded by trained personnel. The EEG will be recorded in a designated room, shielded from outside electrical interference using 25 gel-mounted electrodes arranged according to the international 10–20 system with additional electrooculographic (EOG) and electrocardiographic (ECG) electrodes. The cognitive testing will be performed by trained personnel with experience in neuropsychological testing. The MR scan will be performed on a 3T scanner (Siemens Magnetom Vida, Siemens Healthcare). Actigraphy will be recorded using a wrist-worn actigraph (ScanWatch, Withings).

### 2.6. Outcome measures

Primary outcome:

The goal is to determine the total gamma power, with no concomitant LTS device stimulation, and to assess changes in the gamma power at around 40 Hz measured with power spectral density and EEG SSVEP from baseline (week 0) to post-intervention follow-up (week 26).

1. Null hypothesis: There is no significant difference between treatment and placebo in total gamma power and power spectral density signal-to-noise ratio at baseline (week 0) and follow-up (week 26).
2. Research hypothesis: There is a significant difference between treatment and placebo in total gamma power and power spectral density signal-to-noise ratio at baseline (week 0) and follow-up (week 26).

The statistical analysis plan will be available before the data collection of the study is completed.

Secondary outcomes:

The secondary outcomes include exploratory outcomes which are listed below. All refer to the change from baseline (week 0) to post-intervention follow-up (week 26), and no-intervention follow-up (week 32), unless otherwise specified.

1. Change in ADAS-Cog Plus EF&FA
2. Change in ADCS-ADL
3. Change in MoCA
4. Change in resting-state BOLD-fMRI connectivity in the default mode network (DMN)
5. Change in rs-EEG connectivity encompass alpha-theta with DMN
6. Change in MR spectroscopy NAA/mI-ratio
7. Change in total sleep time and wakefulness after sleep onset
8. Change in blood-based biomarkers of AD (plasma Aβ42/40-ratio, plasma tau181, plasma tau231, plasma neurofilament light chain, and plasma glial fibrillary acidic protein)
9. Change in global atrophy (ventricular volume) and in hippocampal volume
10. Change in MR Arterial Spin labeling (ASL) perfusion in the posterior cingulate gyrus, precuneus, inferior parietal lobe, and lateral prefrontal cortex
11. Change in rs-EEG spectral composition in the dominant alpha rhythm

In addition to these secondary endpoints, safety will be monitored by the number of Adverse Events (AE), and similarly, occurrences of ARIAs will be monitored. The compliance/adherence will be measured directly by the device which includes a camera with facial recognition software. This determines the number of days missed, the time present in front of the device as well as gaze direction to assess the time of direct exposure. Furthermore, compliance and feasibility will be evaluated via a structured interview that includes questions on the perception of flickering, brightness, and usability of the device.

### 2.7. Withdrawal and drop-out

All participants are free to withdraw from the study at any time and are not required to give any reason for doing so. If a participant at any time does not fulfill the in- and exclusion criteria, they will be withdrawn from the study, except if this is solely the progression from moderate to severe AD. The principal investigator may additionally halt the trial for an individual participant or the entire trial, should there be any major safety concerns with the continuation of the study.

In case of drop-outs or missing data for any other reason, a last observation carried forward approach will be used to handle the missing data.

### 2.8. Recruitment and sample size

Participants will be recruited through contact with their usual healthcare professionals on the Danish Island of Zealand with a population of approximately 2.7 million inhabitants (Danmarks Statistik, 2021).

The sample size calculation is based on the primary outcome and has been based on effect sizes reported (Xing et al., 2018; Jones et al., 2019). The estimation is carried out using R version 3.6.3 with “pwr” version 1.2–2 with the following assumptions: (1) Two-sample *T*-test assuming equal variance, (2) Equal allocation, (3) Signal-to-noise (SNR) ratio at 40 Hz of 2.7 dB in the active treatment group and 0 dB in the placebo group at 6 months, (4) Standard deviation of 3.2 dB in both groups, (5) A power of 90%, and (6) Type 1 error of 5%. A 50% drop-out rate is assumed based on previous experience, resulting in a sample size of 124 (62 in each arm).

### 2.9. Statistical methods

The demographics will be reported at exact values for each arm to characterize each arm.

Full completion of all datasets in a clinical trial like this is unlikely; therefore, two different populations will be used for analysis. The first population will be used to assess the effect of the treatment and will be the per protocol population (PPP). The PPP is defined by all data points available for analysis. The other population is the intention to treat (ITT) population. The ITT will be used when assessing safety and feasibility outcomes. This is to ensure that participants who drop-out due to adverse events are being evaluated as part of the study.

### 2.10. Data statement

The investigators must ensure that the personal data of all participants are handled confidentially and that all study data are recorded truthfully, including recording all adverse events. Only study personnel (investigators) will have access to the full data set. Digital data will be stored on encrypted hard drives and stored together with analog data in a locked file cabinet in a locked room.

This trial will be monitored by the regional unit for Good Clinical Practice (GCP-unit) at Frederiksberg Hospital to ensure proper data management and conduct.

## 3. Discussion

The discovery of safe and effective treatment options for patients with AD is crucial, especially treatments that can modify the disease progression. Indeed, the majority of the currently available treatment options mainly produce symptomatic relief (Long and Holtzman, 2019). Numerous studies have been, and are therefore being conducted in an attempt to find potential disease-modifying treatment options (Cummings et al., 2021). This has led to recent advances in treatment using antibodies against Aβ (Reardon, 2023; van Dyck et al., 2023).

Growing evidence has shown a beneficial effect of the induction of 40 Hz neural oscillations in Alzheimer’s disease (Clements-Cortes et al., 2016; Chan et al., 2021; Cimenser et al., 2021). The induction of 40 Hz gamma oscillations through non-invasive exposure to flickering lights is fundamentally different from the many trials using drugs, as it attempts to restore gamma oscillation and is expected to reduce the Aβ and tau load by previously described mechanisms. This may be the paradigm shift necessary to achieve the needed breakthrough (Adaikkan and Tsai, 2020).

Nevertheless, a few aspects must be further discussed. For one, previous studies have already shown the ability of ISF to induce 40 Hz gamma oscillations (Agger et al., 2022). However, while the induced 40 Hz signal of ISF has a similar spatial distribution as the induced signal from stroboscopic light, the power amplitude of ISF has been shown to be lower (Agger et al., 2022). This may increase the risk of the ISF intervention failing to show an effect. It is, nevertheless, not yet certain whether the power of the induced 40 Hz signal, the spatial distribution, the time above a certain threshold, or other undiscovered factors are the main driving mechanisms. The use of ISF is also expected to decrease the discomfort of the participants and increase compliance compared to stroboscopic light. However, this claim is outside of this study’s scope, as a direct comparison between ISF and stroboscopic light would be needed.

Furthermore, the use of 40 Hz ISF and matched continuous white light enables a true placebo-controlled study design, which is expected to greatly enhance the validity of the findings of this study as compared to a visually distinguishable device as used in other studies (Chan et al., 2021; Cimenser et al., 2021) or studies on vibrotactile stimulation without placebo control (Clements-Cortes et al., 2016). This does, however, have some drawbacks as the effect of continuous white light is not perfectly understood, and may therefore also influence the results. Nevertheless, the use of a sham device with an almost true placebo is expected to increase the validity of any positive findings in this study.

Lastly, while this is a relatively large single-center study, it may still be underpowered to show a clinical effect as the arguably most important outcome of a clinical trial in AD is cognitive performance. In this study the ADAS-Cog Plus EF & FA (Skinner et al., 2012) is used which requires a sample size of 547 per group, assuming 80% power and an alpha of 0.05 with an expected effect size of 25%. This is well outside of the scope of this trial. Consequently, cognitive performance has not been chosen as a primary endpoint but rather effects on electrophysiology.

Overall, this trial uses an extensive and multi-disciplinary examination battery including neuroimaging, cognitive testing, electrophysiology, blood based biomarkers and self-reported measurements with several pre-defined outcomes, in order to gain further insight into the neural mechanisms of induced 40 Hz gamma oscillations. In conclusion, this protocol describes a randomized, double-blinded, placebo-controlled, clinical trial which may provide a good indication as to whether induction of 40 Hz gamma oscillations is a possible new treatment for AD.

## Ethics statement

This trial will test a possible novel treatment for AD with a very limited risk of harm to the study participants. If a study participant experiences any adverse events requiring medical treatment, this will be covered by the national medical insurance. This project has been approved by the Danish Medical Agency (ID: 2021070345, CIV-21-09-037667, Nov-23-2021) and the Danish Scientific Ethics Committee (DKETIK 2113349, Nov-19-2021). All methods will be carried out in accordance with relevant guidelines and regulations. Informed consent will be given by all included participants. Participants are free to leave the trial at any point with no consequence to their medical treatment outside of the trial.

## Author contributions

MC, TK, and PP initiated the project. MA, MH, ED, and MC wrote the majority of the clinical investigation plan. MA, MH, and ED obtained regulatory approvals. MA, MH, ED, and MC prepared the manuscript. AB, PP, PH, MN, KHM, and KM provided significant technical and clinical support to the design of the study. All authors contributed to the article and approved the submitted version.

## Figures and Tables

**Table 1:** 

AD	Alzheimer’s disease
ADAS-Cog Plus EF&FA	Alzheimer’s Disease Assessment Scale-Cognitive Subscale Plus Executive Function & Functional Ability
ADSC-ADL	Alzheimer’s Disease Cooperative Study – Activities of Daily Living
AE	Adverse Events
ARIA	Amyloid-related imaging abnormalities
ASL	Arterial Spin Labeling
BOLD-fMRI	functional Blood Oxygen Level Dependent Magnetic Resonance Imaging
DMN	Default Mode Network
EMA	European Medical Agency
EEG	Electroencephalography
EOG	Electrooculography
ECG	Electrocardiographic
GCP	Good Clinical Practice
ITT	Intention to Treat
ISF	Invisible Spectral Flicker
LTS	Light Therapy System
MoCA	Montreal Cognitive Assessment
MRI	Magnetic Resonance Imaging
NIA-AA	National Institute on Aging and Alzheimer’s Association
PPP	Per Protocol Population
rs-EEG	Resting-state Electroencephalography
SAE	Serious Adverse Avent
SSVEP	Steady State Visual Evoked Potential
SNR	Signal-to-noise
TMS	Transcranial Magnetic Stimulation
